# CanFlyet: habitat zone and diet trait dataset for Diptera species of Canada and Greenland

**DOI:** 10.3897/BDJ.12.e129610

**Published:** 2025-03-25

**Authors:** Samantha E Majoros, Tyler A Elliott, Sarah J. Adamowicz

**Affiliations:** 1 University of Guelph, Guelph, Canada University of Guelph Guelph Canada; 2 Biodiversity Institute of Ontario, Guelph, Guelph, Canada Biodiversity Institute of Ontario, Guelph Guelph Canada

**Keywords:** Diptera, Canada, Greenland, dataset, habitat, diet, ecological traits

## Abstract

**Background:**

Flies (Diptera) are an ecologically important group that play a role in agriculture, public health and ecosystem functioning. As researchers continue to investigate this order, it is beneficial to link the growing occurrence data to biological traits. However, large-scale ecological trait data are not readily available for fly species. While some databases and datasets include fly data, many ecologically relevant traits for taxa of interest are not included. In this study, we create a dataset containing ecological traits (habitat and diet) for fly species of Canada and Greenland having occurrence records on the Barcode of Life Data Systems (BOLD). We present a dataset containing trait information from the literature for 981 Diptera species.

**New information:**

Diptera species were chosen for the dataset, based on the occurrence records available for Diptera species from Canada and Greenland on the Barcode of Life Data System (BOLD). Trait data were then compiled and digitised in a standardised format, based on 667 works from literature published before April 2024. Traits were assigned at the lowest taxonomic level available. Three biological traits were included: larval habitat, larval diet type and adult diet. The dataset contains traits for 981 species across 380 genera, 34 subfamilies and 61 families. This dataset allows for assignment of traits to occurrence data for Diptera species and can be used for further research into the ecology, evolution and conservation of this order.

## Introduction

Flies (Diptera) are a diverse, widespread taxonomic group, occurring across a wide range of ecological niches and geographic regions (Marshall 2012,Adler and Courtney 2019). This important group occupies various ecological roles, many of which have an impact on the environment and agricultural practices. Fly species can act as pests to crops, such as *Liriomyzabryoniae* on cabbages, lettuce and tomatoes (Bragard et al. 2020); *Deliaradicum* on broccoli and cauliflower (Mesmin et al. 2019); *Dasineuramali* on apples (Wearing et al. 2013); and *Bradysiaocellaris* and *Lycoriellaingenua* on mushrooms (Shamshad 2010). Diptera also includes pests of livestock, such as the blood-feeding *Stomoxyscalcitrans* (Olafson et al. 2021). Flies are also a group of interest regarding public health, as some, like *Calliphoravomitoria*, are vectors of pathogenic microorganisms (Bedini et al. 2017). Despite these detrimental examples, flies also provide many important ecological benefits. Flies are an important pollinator group, especially in Arctic and northern environments (Tiusanen et al. 2016). The family Muscidae includes species that are pollinators of key plant species in these regions and play an important role in the ecosystem (Tiusanen et al. 2016). Flies also fill various other ecological roles, such as parasitoids of other insects that act as vectors for pathogenic bacteria (Molinatto et al. 2020). Many species act as ecosystem engineers; changing environments through suspension-feeding, grazing, burrowing and predation, as well as serving as an important food source for other organisms (Adler and Courtney 2019). The saprophagous diet of many flies is also important for the breakdown of decaying organic material (Adler and Courtney 2019). As biocontrol agents and bioindicators of water quality, flies act as a valuable resource for food production and research (Adler and Courtney 2019).

This ecologically important group has been the focus of large DNA barcoding efforts, such as the Global Malaise Trap Program, which seeks to document Diptera diversity (which is likely under-described) and open doors for ecological research and monitoring (Geiger et al. 2016, deWaard et al. 2018, Global Malaise Trap Program 2023). DNA barcoding involves using a standardised gene region to identify and delineate species (Hebert et al. 2003) and there is a large amount of DNA barcoding data available on databases such as the Barcode of Life Data System (BOLD) (Ratnasingham and Hebert 2007), which contained 6,660,909 Diptera records representing 36,125 species as of 2 Oct 2024. There is also a large amount of species occurrence data on databases such as the Global Biodiversity Information Facility (GBIF) (GBIF.org 2024), which contained 24,618,171 occurrences as of 2 Oct 2024. As Diptera research continues, it is valuable to link such occurrence records with biological data.

However, finding biological trait data for fly species can be a challenge. There are various databases or datasets currently available for Diptera and other insect groups, covering a varying number of traits and taxa (Table 1). However, few databases focus solely on Diptera and, while flies are included in some databases, not all species and traits of interest are included. A larger focus also appears to be on morphological traits, such as body size, with information on ecological traits, such as habitat and diet, being less readily available. Finally, few databases focus on Diptera from Canada or Greenland. Overall, traits for many Diptera species are very difficult to find and, when known, are typically present in taxon-specific studies and prose format rather than compiled datasets with standardised digitisation.

The goal of this study was to create a dataset containing ecological traits (habitat and diet type) for selected Diptera species found in Canada and Greenland. These traits were selected due to the paucity of such information in available datasets and the value of this information for understanding shifts in traits and not only taxonomic composition, across space and time. Species were selected that have multiple occurrence records and high-quality DNA barcode sequences on BOLD (following criteria outlined in Majoros et al. (2023)), which likely includes many of the most common species in the study region. This trait dataset is expected to be of particular relevance for species caught in Malaise traps, given the extensive usage of this collection modality in large-scale DNA barcoding efforts. This dataset can be used in future research to further our knowledge and understanding of this important taxonomic group, including ecology and temporal studies of insect communities with climate and land-use changes.

## General description

### Purpose

This study provides a dataset containing the larval habitat and adult and larval diet categories for Diptera species of Canada and Greenland having occurrence records on BOLD. The traits were assigned to taxa using the currently available literature. This dataset will aid in further research that involves the ecological traits of Diptera species.

## Project description

### Title

CanFlyet: Habitat Zone and Diet Trait Dataset for Diptera Species of Canada and Greenland

### Personnel

Samantha E. Majoros, Tyler A. Elliott and Sarah J. Adamowicz

### Design description

The dataset provides larval habitat, larval diet and adult diet categories for Diptera species found in Canada and Greenland. Traits were determined based on a literature search.

### Funding

Funding support for this project comes from the Natural Sciences and Engineering Research Council of Canada; the Government of Canada through Genome Canada and Ontario Genomics; the Ontario Ministry of Economic Development, Job Creation and Trade; and Food from Thought: Agricultural Systems for a Healthy Planet Initiative programme funded by the Government of Canada through the Canada First Research Excellence Fund.

## Sampling methods

### Study extent

The fly species were chosen for inclusion in this dataset by first downloading data for Diptera from Canada and Greenland from BOLD directly into R using BOLD’s application programming interface (API) on 24 June 2021. This dataset was originally used in Majoros et al. (2023), in which species were represented by Barcode Index Numbers (BINS), which are operational taxonomic units (OTUs) used by BOLD that are clusters of barcode sequences similar to species (Ratnasingham and Hebert 2013). In addition to BINs, the analysis was also repeated by clustering records by a 4% clustering threshold and using these clusters in the place of BINs. Studies have shown that a 3-5% clustering threshold is suitable for clustering within the Diptera family Chironomidae (Lin et al. 2015, Baloğlu et al. 2018). The records were filtered based on the requirements outlined in Majoros et al. (2023), which included that records needed to possess high-quality DNA sequence data and be identified to the species or genus level, that BINs and clusters needed to possess at least 20 records and be found in at least two geographic regions meeting the requirements of Majoros et al. (2023) and needed to be represented by at least 10 records in each region in which they were found. The remaining species represented by these BINs and clusters were chosen for analysis and inclusion in this dataset. The traits were assigned to taxa using a series of literature searches conducted between April 2021 and April 2024. The dataset was formatted using the DarwinCore format (Darwin Core Maintenance Group 2023).

### Sampling description

The traits included in this dataset are larval habitat and adult and larval diet categories. These traits were chosen due to their importance in ecological functioning as well as the impact these traits can have on species distributions and population genetic structure. These traits were used in Majoros et al. (2023) as part of the case study. The biological traits for each species were determined and assigned through literature searches conducted from April 2021 to April 2024. Through the Omni Academic search tool available through the University of Guelph and Google Scholar, traits were found using the following search terms: trait AND “Taxonomic name”, habitat AND “Taxonomic name”, diet AND “Taxonomic name”, “Feeding mode” AND “Taxonomic name”, biology AND “Taxonomic name”, “Natural history” AND “Taxonomic name”, “Life history” AND “Taxonomic name”, taxonomy AND “Taxonomic name”, catalogue AND “Taxonomic name” and “Field guide” AND “Taxonomic name”. This dataset compiles information from 667 published sources. The title and DOI of each reference are included in the dataset and the full references are available in Suppl. material 1. Traits were assigned to the lowest taxonomic level possible; however, not all traits could be assigned at the species level. For these species, traits were assigned using data from the next lowest level available, whether genus, subfamily or family. For example, if the authors of a given study mentioned that a particular genus has a specific trait, then species belonging to that genus were assigned that trait. The taxonomic level of the trait data is included in the dataset.

For larval habitat, species were classified as terrestrial (defined as taxa that live primarily in land habitats), aquatic (defined as taxa that live primarily in waterbodies or associated habitats) or semi-aquatic (defined as species that primarily live in wet habitats and require high levels of moisture and some elements of both terrestrial and aquatic habitats). These assignments were made based on the larval habitat requirements. For adult and larval diet, species could be classified as predaceous (taxa that prey on insects or other animals), mycophagous (those that feed on fungi), saprophagous (those that feed on decaying organic matter), nectar/pollen/honeydew feeding (taxa that primarily feed on nectar, pollen and/or honeydew), parasitic (those that live and feed in or on an organism of another species), parasitoid (organism whose young develop within a host, eventually resulting in host death), leaf/root/stem feeding (those that feed on the leaves, roots and/or stems of plants), detritus and algae feeding (those that feed on small particles of algae and detritus), kleptoparasitic (those that take food and resources from another species), polyphagous (those that engage in more than three of the other feeding modes), non-feeding (those that do not feed at the specified life stage) and unclear (taxa for which there is not enough information to make a trait assignment). Taxa can also be assigned a combination of the diet categories. Many higher taxonomic levels are diverse and contain species with different traits. In this case, the most common category was used if specific information for a species was not available. When this was unclear, multiple categories were included separated by “or”.

### Quality control

The traits in the dataset were assigned on the basis of reading the literature by a single researcher. To check the trait assignments for accuracy and consistency, the dataset was sent to another researcher for review. This second researcher randomly selected 20 species from the dataset and reviewed the referenced literature within the dataset for the species to ensure that the trait assignments were accurate and consistent. Knowledge of fly traits is likely to develop as further research is conducted and we are open to suggestions from the community, via comments on the manuscript, dataset or GitHub and will update the dataset as needed.

## Geographic coverage

### Description

This dataset includes Diptera species found in Canada and Greenland that also have multiple occurrence records and DNA barcode sequences publicly available on BOLD. Ten of the species are unique to Greenland and the rest are found in Canada.

## Taxonomic coverage

### Description

**Figure 1.**
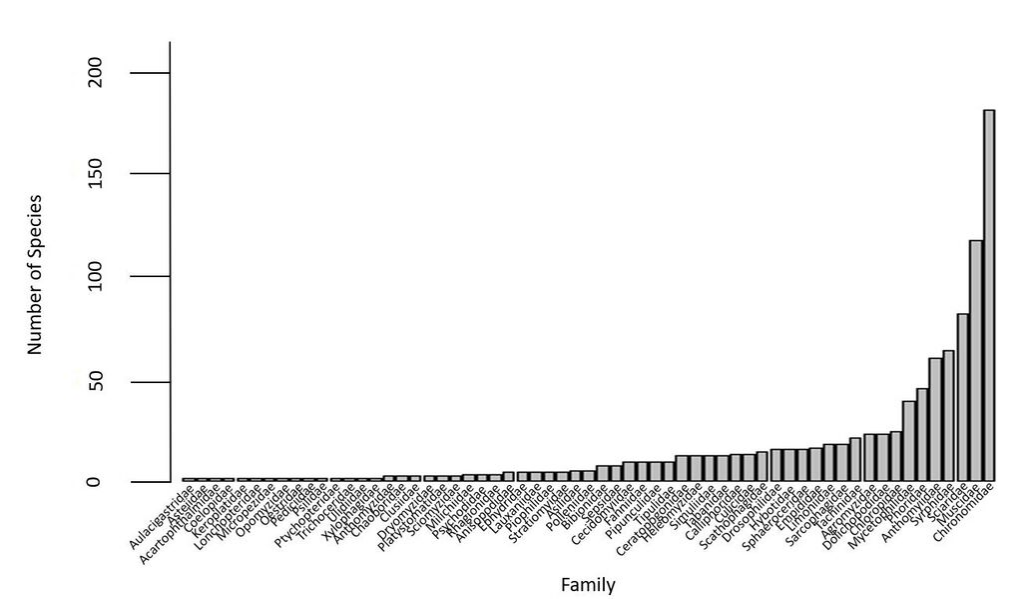
The number of Diptera species from Canada and Greenland of each family that is present in the dataset. The selected species included here also have multiple occurrence records and DNA barcode sequences publicly available on BOLD. For several families, only one species is included. Chironomidae is the largest family within the dataset, with 182 species present.

The dataset contains traits for 981 species across 380 genera, 34 subfamilies and 61 families. The families are represented by varying numbers of species within the dataset (Fig. 1). For several families, the dataset only contains information for one species. The most species-rich family in this dataset is Chironomidae, which is represented by 182 species.

## Traits coverage

**Figure 2.**
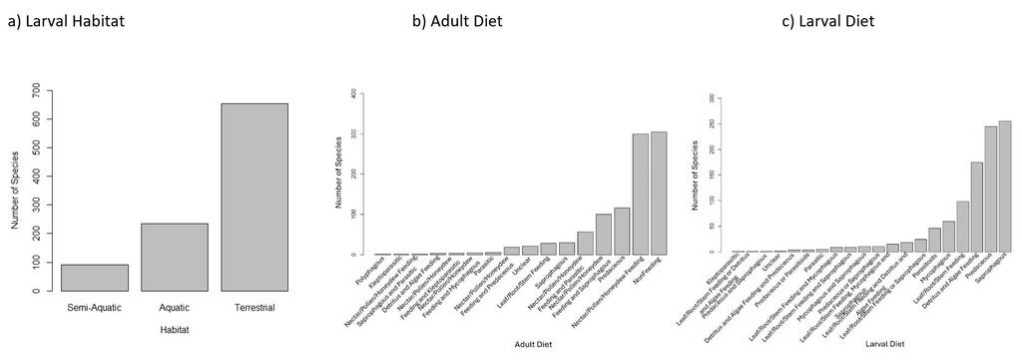
The number of Diptera species from Canada and Greenland in this dataset having each trait category for: **a** larval habitat; **b** adult diet; **c** larval diet. The majority of species included in the dataset are terrestrial. Most species are non-feeding at the adult life stage and few are polyphagous or kleptoparasitic as adults. The majority of species included in the dataset are saprophagous as larva and few are kleptoparasitic.

The dataset contains information for three biological traits: larval habitat, adult diet and larval diet. For larval habitat, 655 species were considered terrestrial, 277 were aquatic and 58 were semi-aquatic (Fig. 2a). The number of species with each adult diet can be found in Fig. 2b and the number of species with each larval diet can be found in Fig. 2c. Traits were assigned to the lowest taxonomic level possible and species-level traits were found for 190 species.

## Temporal coverage

### Notes

The literature searches were conducted from April 2021 to April 2024. The papers referenced were published from 1889 to 2023.

## Usage licence

### Usage licence

Creative Commons Public Domain Waiver (CC-Zero)

## Data resources

### Data package title

CanFlyet: Habitat Zone and Diet Trait Dataset for Diptera Species of Canada and Greenland

### Number of data sets

1

### Data set 1.

#### Data set name

CanFlyet: Habitat Zone and Diet Trait Dataset for Diptera Species of Canada and Greenland

#### Data format

csv, xlsx, tsv

#### Download URL

https://datadryad.org/stash/dataset/doi:10.5061/dryad.fqz612jwx

#### Data format version

xlsx is version 2450. Csv and tsv are UTF-8.

#### Description

The dataset presents larval habitat, adult diet and larval diet for 981 species across 380 genera, 34 subfamilies and 61 families. A table showing the column labels and descriptions is provided in below. The Diptera species included are from Canada and Greenland and have multiple occurrence records and DNA sequence data on BOLD. The traits were assigned to taxa using a series of literature searches conducted between April 2021 and April 2024. This dataset can be used by researchers to determine biological traits for Diptera species and conduct research that involves ecological trait data.

**Data set 1.** 

Column label	Column description
id	The serial number of the record.
taxonRank	The taxonomic level the record has been identified to.
order	The order-level taxonomic classification.
family	The family-level taxonomic classification.
subfamily	The subfamily-level taxonomic classification.
genus	The genus-level taxonomic classification.
specificEpithet	The species-level taxonomic classification.
scientificName	The scientific name of the species.
habitat	The larval habitat in which the taxon is found. Taxa can be assigned terrestrial (taxa that live primarily in land habitats), aquatic (taxa that live primarily in waterbodies or associated habitats) or semi-aquatic (taxa that primarily live in wet habitats and require high levels of moisture and some elements of both terrestrial and aquatic habitats).
traitName	The name of the biological trait. The traits included are adultDiet and larvalDiet.
traitValue	The trait category assigned to the species. Taxa can be assigned predaceous (taxa that prey on insects or other animals), mycophagous (those that feed on fungi), saprophagous (those that feed on decaying organic matter), nectar/pollen/honeydew feeding (taxa that primarily feed on nectar, pollen and/or honeydew), parasitic (those that live and feed in or on an organism of another species), parasitoid (organism whose young develop within a host, eventually resulting in host death), leaf/root/stem feeding (those that feed on the leaves, roots and/or stems of plants), detritus and algae feeding (those that feed on small particles of algae and detritus), kleptoparasitic (those that take food and resources from another species), polyphagous (those that engage in more than three of the other feeding modes), non-feeding (those that do not feed at the specified life stage) and unclear (taxa for which there is not enough information to make a trait assignment). The taxa can also be assigned a combination of these categories. In this case, the categories are separated by “and”. For example, “saprophagous and predaceous”. For cases where it is unclear which diet is more common, the categories are separated by “or”. For example, “saprophagous or predaceous”.
traitAssignmentLevelHabitat	The taxonomic level from which the habitat assignment was obtained and assigned.
traitAssignmentLevelAdultDiet	The taxonomic level from which the adult diet assignment was obtained and assigned.
traitAssignmentLevelLarvalDiet	The taxonomic level from which the larval diet assignment was obtained and assigned.
notes	Additional information on the habitat and diet of the taxa. Information is provided for the lowest taxonomic level used for the trait assignments if available.
basisOfRecord	From where the trait data were obtained.
Number_of_references_consulted	The number of sources referenced when obtaining the trait information for each taxon.
references	The title and DOI of the reference used for the trait assignment. Full references are included in the manuscript.

## Additional information

For this study, we created a dataset containing larval habitat and adult and larval diet for 981 Diptera species across 61 families found in Canada and Greenland. This dataset also contains trait information for nine taxa only identified to the genus level. This dataset allows for assignment of traits to a large variety of species. This is a valuable resource for bioinformatics work, as well as for ecological studies. This dataset was originally used to determine the relationship between biological traits and population genetic structure (Majoros et al. 2023) and can be applied to a wide range of other studies and research. These traits can also be linked to other types of data, such as species occurrence or community composition data.

The species included in this dataset possessed a wide variety of different habitat and dietary requirements. Most species were terrestrial, non-feeding at the adult life stage or saprophagous at the larval stage. Other diets were far less represented, such as those with a kleptoparasitic life stage.

**Figure 3.**
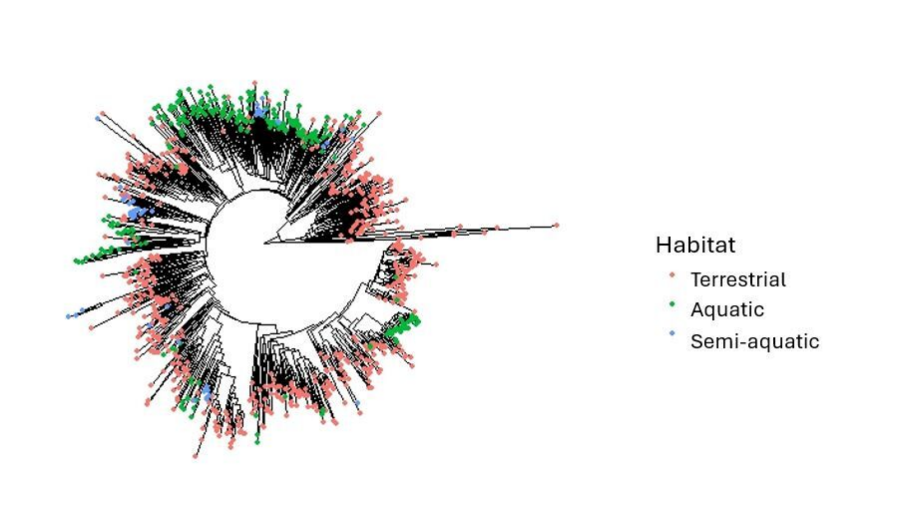
Phylogenetic tree of Diptera species from Canada and Greenland included in this dataset; these species also have multiple occurrence records and DNA barcode sequences publicly available on BOLD. The different colours of the tips represent species with different larval habitat categories. The traits provided in the dataset could be used to investigate the relationship between traits and evolutionary history. These Maximum Likelihood trees were created using cytochrome c oxidase subunit 1 (CO1) sequences from BOLD and functions from the package phangorn version 2.11.1 (Schliep 2011) in the R programming language (R Core Team 2023). The tips were coloured using the package ggtree version 3.6.2 (Yu et al. 2017). The code for how to create this tree is provided at https://github.com/S-Majoros/Diptera_Dataset_Phylogenetic_Tree.

**Figure 4.**
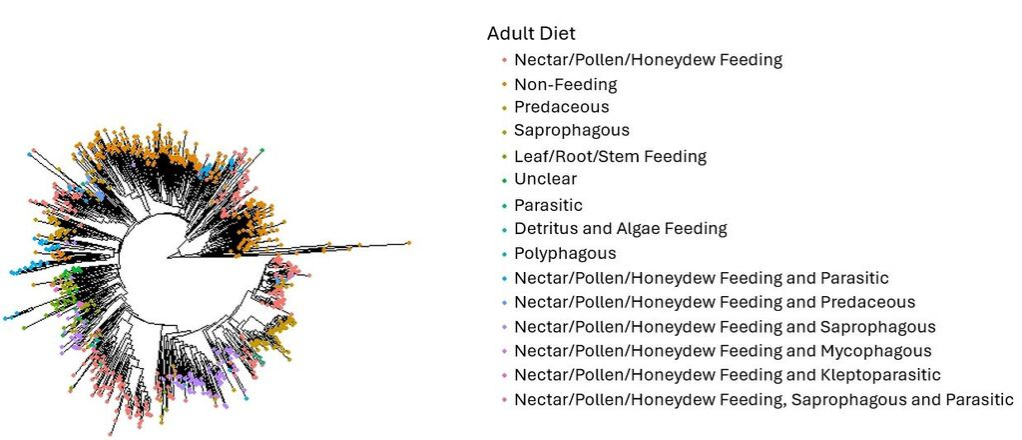
Phylogenetic tree of Diptera species from Canada and Greenland included in this dataset; these species also have multiple occurrence records and DNA barcode sequences publicly available on BOLD. The different colours of the tips represent species with different adult diet categories. The traits provided in the dataset could be used to investigate the relationship between traits and evolutionary history. These Maximum Likelihood trees were created using cytochrome c oxidase subunit 1 (CO1) sequences from BOLD and functions from the package phangorn version 2.11.1 (Schliep 2011) in the R programming language (R Core Team 2023). The tips were coloured using the package ggtree version 3.6.2 (Yu et al. 2017). The code for how to create this tree is provided at https://github.com/S-Majoros/Diptera_Dataset_Phylogenetic_Tree.

**Figure 5.**
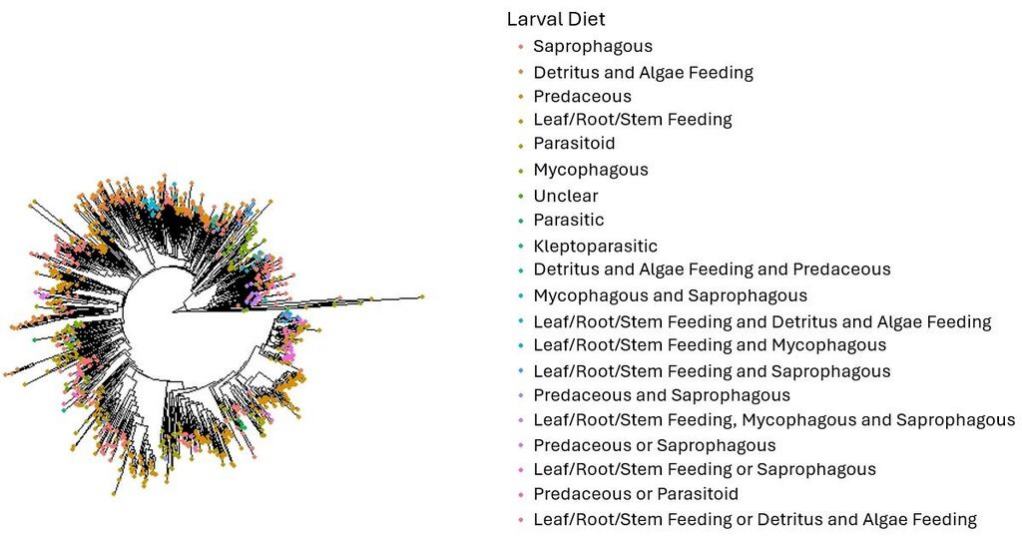
Phylogenetic tree of Diptera species from Canada and Greenland included in this dataset; these species also have multiple occurrence records and DNA barcode sequences publicly available on BOLD. The different colours of the tips represent species with different larval diet categories. The traits provided in the dataset could be used to investigate the relationship between traits and evolutionary history. These Maximum Likelihood trees were created using cytochrome c oxidase subunit 1 (CO1) sequences from BOLD and functions from the package phangorn version 2.11.1 (Schliep 2011) in the R programming language (R Core Team 2023). The tips were coloured using the package ggtree version 3.6.2 (Yu et al. 2017. The code for how to create this tree is provided at https://github.com/S-Majoros/Diptera_Dataset_Phylogenetic_Tree.

Trait data have been used to investigate various ecological and evolutionary patterns and topics, such as community assembly (Kraft and Ackerly 2010, Zhang et al. 2020), molecular evolution (May et al. 2020), phylogenetic assemblage structure (Barnagaud et al. 2014) and how populations change over time or in response to ecological change (De Palma et al. 2015, Coulthard et al. 2019). Similarly, this dataset can be used to answer a variety of questions and is a useful tool for future research. It can be used for studies that relate to ecology, evolution, biogeography and conservation of fly species and include the habitat and diet of flies. The dataset can be used to investigate the relationship between traits and population genetic structure or phylogenetic community structure. The dataset could also be used to find relationships between traits, such as in studies like Freire et al. (2021). Visualisations are provided in Fig. 3, Fig. 4 and Fig. 5 of the phylogenetic relationships between species possessing different traits. This approach could be built upon to look at the relationship between traits and evolutionary history, such as in studies like Rainford and Mayhew (2015). The trait dataset can also be used to investigate the diversity of different fly traits in an area of interest, to determine how these traits impact occurrence patterns, how traits may relate to responses to environmental change or to determine which species may be most likely to colonise these areas in the future. Trait data could also prove useful for biomonitoring of insect diversity in agricultural areas, such as research into which farming practices are associated with a balance of ecological functions in associated insect communities.

While this dataset provides useful trait information for a large number of Diptera species, more research is needed to expand our knowledge of this ecologically important group. This dataset can be expanded upon to include other important traits, such as adult habitat, more detail about foods comprising the larval and adult diets, reproductive traits, mobility traits and vector status. With the inclusion of additional traits, the dataset can be used to answer an even larger variety of biological questions. It is also important to consider the diversity of Diptera species and we note that, even within genera, species may differ in habitat and diet requirements. While we made trait assignments based on previous research and taxonomy, further research should be done to explore uncertainties, add detail and achieve species-level data. Diptera habitat preferences can also vary across study areas and further research should be done to understand the ecology of Diptera species in any given geographic area. While it is hard to capture these details in a general dataset, the data provided in this study provide a valuable resource for a range of scientific studies, as well as a building block for future work.

In summary, we share a trait dataset containing habitat and diet information for Diptera taxa from Canada and Greenland that also have multiple occurrence records and DNA barcoding sequences available on BOLD. This dataset is publicly available on Data Dryad (https://datadryad.org/stash/dataset/doi:10.5061/dryad.fqz612jwx) and is also available on GitHub (https://github.com/S-Majoros/Diptera_Dataset_Phylogenetic_Tree). The file is available in csv, xlsx and tsv formats in order to make it as accessible as possible for future users. This dataset is publicly available so that other researchers can use it to conduct further studies, answer more questions and improve our knowledge of this ecologically important group.

## Supplementary Material

22971C70-2D1B-519D-ACF5-C835C6EA31A3 10.3897/BDJ.12.e129610.suppl1 Supplementary material 1 References for Diptera habitat and diet traits Data type Literature References Brief description This file contains the references used to determine habitat and diet for Diptera species from Canada and Greenland. File: oo_1061654.pdf https://binary.pensoft.net/file/1061654 Samantha E. Majoros, and Sarah J. Adamowicz

## Figures and Tables

**Table 1.** Overview of the publicly available databases and datasets that include insects. This table includes databases and datasets that were publicly available as of 2 Feb 2023 and were found through a literature search. Number of records are as of 2 Feb 2023.

Database Name	Taxonomic Focus	Geographic Focus	Types of Traits	Number of Traits	Number of Records	Data Availability	References
DISPERSE*^1^	Annelida, Mollusca, Platyhelminthes & Arthropoda	Europe	Dispersal	9	480	Download as xlsx file	Sarremejane et al. (2020)
Freshwater Biological Traits*^2^	Freshwater macroinvertebrate taxa	North America	Morphology, life history, mobility, environmentaltolerance, resource acquisition/ preference	26	11912	Select taxa, region and traits on website. Download as an xlsx file	United States Environmental Protection Agency (2012)
Animal Traits*^3^	Arthropoda, Chordata, Mollusca and Annelida	Global	Body mass, metabolic rate, brain size	3	3580	Download xlsx or csv file	Herberstein et al. (2022)
CONUS*^4^	Arthropoda	United States of America	Life history, dispersal, morphology, ecology	11	2.05 million	Download csv file	Twardochleb et al. (2021)
Lotic Invertebrate Traits for North America*^5^	Invertebrates	North America	Ecology, morphology, behaviour, physiology	62	14127	Download as a txt file	Vieira et al. (2006)
European & Maghreb Butterfly Trait Database*^6^	Lepidoptera	Europe and North Africa	Life history, morphology, resource-based, behaviour	25	542	Download as xlsx file	Middleton-Welling et al. (2020)
GlobalAnts*^7^	Formicidae	Global	Morphology, ecology, life history	23	82910	Need to make an account to access. Can search for desired traits and download as a csv file	Parr et al. (2016)
The Odonate Phenotypic Database*^8^	Odonata	Global	Morphology, life history, behaviour	35	3978	Download as a xlsx file	Waller et al. (2019)
Morphological trait database of saproxylic beetles*^9^	Saproxylic Coleoptera	Europe	Morphology	13	1376	Download as a csv file	Hagge et al. (2021)
Data from: Sensitivity of functional diversity metrics to sampling intensity*^10^	Carabidae	The Netherlands	Morphology	7	73	Download as txt file	van der Plas et al. (2017)
Data from: A summary of eight traits of Coleoptera, Hemiptera, Orthoptera and Araneae, occurring in Grasslands in Germany*^11^	Arthropoda	Germany	Morphology, ecology	8	1230	Download as txt file	Gossner et al. (2015)
LepTraits*^12^	Lepidoptera	Global	Morphology, habitat, reproduction, hostplant association	6	75103	Download as csv file	Shirey et al. (2022)
The Insect Trait Tool (ITT)*^13^	Arthropoda	Germany	Habitat, Diet	2	34085	Download as a pdf or .xlsx file	Hörren et al. (2022)

## Footnotes

*1
https://figshare.com/articles/dataset/DISPERSE_a_trait_database_to_assess_the_dispersal_potential_of_European_aquatic_macroinvertebrates/12417251/1

*2
https://cfpub.epa.gov/ncea/global/traits/search.cfm

*3
https://animaltraits.org/

*4
https://portal.edirepository.org/nis/mapbrowse?packageid=edi.481.5

*5
https://pubs.er.usgs.gov/publication/ds187

*6
https://butterflytraits.github.io/European-Butterfly-Traits/index.html

*7
http://globalants.org/

*8
http://www.odonatephenotypicdatabase.org/shiny/odonates/?_inputs_&choose_species=%22%22

*9
https://datadryad.org/stash/dataset/doi:10.5061/dryad.2fqz612p3

*10
https://datadryad.org/stash/dataset/doi:10.5061/dryad.1fn46

*11
https://datadryad.org/stash/dataset/doi:10.5061/dryad.53ds2

*12
https://springernature.figshare.com/collections/LepTraits_1_0_A_globally_comprehensive_dataset_of_butterfly_traits/5899187/1

*13
https://www.biorxiv.org/content/10.1101/2022.01.25.477751v1.full
